# Rapid imaging of intravenous gadolinium-based contrast agent (GBCA) entering ventricular cerebrospinal fluid (CSF) through the choroid plexus in healthy human subjects

**DOI:** 10.1186/s12987-024-00571-3

**Published:** 2024-09-16

**Authors:** Yuanqi Sun, Di Cao, Jay J. Pillai, Adrian Paez, Yinghao Li, Chunming Gu, Jacob M. Pogson, Linda Knutsson, Peter B. Barker, Peter C. M. van Zijl, Arnold Bakker, Bryan K. Ward, Jun Hua

**Affiliations:** 1 F.M. Kirby Research Center for Functional Brain Imaging, Kennedy Krieger Institute, 707 N Broadway, Baltimore, MD 21205 USA; 2 Neurosection, Division of MRI Research, Russell H. Morgan Department of Radiology and Radiological Science, Johns Hopkins University School of Medicine, Baltimore, MD USA; 3 Department of Biomedical Engineering, Johns Hopkins University, Baltimore, MD USA; 4 Division of Neuroradiology, Mayo Clinic College of Medicine and Science, Rochester, MN USA; 5 Department of Neurosurgery, Johns Hopkins University School of Medicine, Baltimore, MD USA; 6 Department of Otolaryngology - Head and Neck Surgery, Johns Hopkins University School of Medicine, Baltimore, MD USA; 7 Department of Neurology, Johns Hopkins University School of Medicine, Baltimore, MD USA; 8 Department of Psychiatry and Behavioral Sciences, Johns Hopkins University School of Medicine, Baltimore, MD USA

**Keywords:** Blood-CSF-barrier, BCSFB, Lymphatic, Partial-volume, Contrast, MRI

## Abstract

**Background:**

Pathways for *intravenously* administered gadolinium-based-contrast-agents (GBCAs) entering cerebrospinal-fluid (CSF) circulation in the human brain are not well-understood. The blood-CSF-barrier (BCSFB) in choroid-plexus (CP) has long been hypothesized to be a main entry-point for intravenous-GBCAs into CSF. Most existing studies on this topic were performed in animals and human patients with various diseases. Results in *healthy* human subjects are limited. Besides, most studies were performed using MRI methods with limited temporal resolution and significant partial-volume effects from blood and CSF.

**Methods:**

This study employs the recently developed dynamic-susceptibility-contrast-in-the-CSF (cDSC) MRI approach to measure GBCA-distribution in the CSF immediately and 4 h after intravenous-GBCA administration in healthy subjects. With a temporal resolution of 10 s, cDSC MRI can track GBCA-induced CSF signal changes during the bolus phase, which has not been investigated previously. It employs a long echo-time (TE = 1347 ms) to suppress tissue and blood signals so that pure CSF signal is detected with minimal partial-volume effects. GBCA concentration in the CSF can be estimated from cDSC MRI. In this study, cDSC and FLAIR MRI were performed immediately and 4 h after intravenous GBCA administration in 25 healthy volunteers (age 48.9 ± 19.5 years; 14 females). Paired t-tests were used to compare pre-GBCA and post-GBCA signal changes, and their correlations with age were evaluated using Pearson-correlation-coefficients.

**Results:**

At ~ 20 s post-GBCA, GBCA-induced cDSC signal changes were detected in the CSF around CP (ΔS/S = − 2.40 ± 0.30%; P < .001) but not in the rest of lateral ventricle (LV). At 4 h, significant GBCA-induced cDSC signal changes were observed in the entire LV (ΔS/S = − 7.58 ± 3.90%; P = .002). FLAIR MRI showed a similar trend. GBCA-induced CSF signal changes did not correlate with age.

**Conclusions:**

These results provided direct imaging evidence that GBCAs can pass the BCSFB in the CP and enter ventricular CSF *immediately* after *intravenous* administration in *healthy* human brains. Besides, our results in healthy subjects established a basis for clinical studies in brain diseases exploiting GBCA-enhanced MRI to detect BCSFB dysfunction.

**Supplementary Information:**

The online version contains supplementary material available at 10.1186/s12987-024-00571-3.

## Background

In recent years, Gadolinium-based contrast agents (GBCAs) enhanced MRI has been widely used and is often considered the gold standard for studying cerebrospinal fluid (CSF) circulation and brain clearance and the glymphatic system in the human brain [1–10]. Since its first approval for human use by the Food and Drug Administration (FDA) in 1988, GBCA enhanced MRI has been an essential component of various routine clinical MRI examinations. According to the American College of Radiology (ACR) [11], macrocyclic GBCAs, the most commonly used type of GBCAs at present, have an excellent safety profile compared to linear GBCAs that were developed and used from the early 1990s. A number of human studies on CSF circulation and brain clearance using GBCAs employed intrathecal injection usually via a lumbar puncture to deliver GBCAs directly into the CSF in the brain [1–5]. Meanwhile, intravenous administration remains the only FDA-approved procedure for GBCA administration in human subjects mainly due to its minimal invasiveness. GBCAs are generally considered intravascular contrast agents when injected intravenously. Intravenous GBCAs largely remain within the blood pool protected by an intact blood brain barrier (BBB) expected to be impermeable to GBCAs. However, GBCA can be detected using inductively coupled plasma mass spectrometry (ICP-MS) in human CSF samples obtained from lumbar puncture in subjects with intact BBB approximately 1–8 h after receiving intravenous GBCAs [12, 13]. Accumulating evidence has been demonstrated that intravenous GBCAs can enter the CSF circulation in the human brain at multiple locations with relatively loose barriers at the interface between blood and CSF. To date, however, the pathways for *intravenous* GBCAs to enter the CSF circulation in the brain remain poorly understood. The current study attempts to investigate one of the potential entry points in the human brain.

It has long been hypothesized that the blood-CSF barrier (BCSFB) in the choroid plexus (CP) [14–18] may be a main site for intravenous GBCAs to enter the CSF circulation [19]. The capillaries in CP are fenestrated, and the BCSFB forms the boundary between the CP and ventricle (CSF) with a monolayer of choroidal epithelial cells with tight junctions that are more permeable than those in typical BBB [14, 15]. Jost et al. [20, 21] has demonstrated in a rat model using both MRI and ICP-MS that GBCA can be detected in the CSF shortly (< 10 min) following intravenous administration of both linear and macrocyclic GBCAs. In human cancer patients, signal enhancement on fluid-attenuated inversion recovery (FLAIR) MR images can be detected in the CP three hours after intravenous injection of GBCAs [7]. To date, however, most studies on intravenous GBCA distribution across the BCSFB were performed in rodent models [20–31] and canine models [32], and in human patients with various brain diseases [7, 9, 33–40]. Since the permeability of BCSFB and BBB may differ drastically between animals and humans, and their integrity may be compromised in brain diseases, our knowledge about how intravenous GBCAs distribute around the CP in *healthy* human subjects is still quite limited. A few imaging studies have demonstrated intravenous GBCA induced signal enhancement in the CP and ventricular CSF in healthy human subjects [7, 10, 35, 41], but several other MRI studies reported seemingly contradictory results [9, 42]. The discrepancies among these studies may be related to several technical challenges for measuring GBCA-induced MR signal changes in the CSF around small structures such as the CP when the GBCA concentration in the CSF is much lower than the blood vessels nearby. The MRI approaches employed in most existing studies have significant signal contributions from both blood and CSF. Therefore, the overall MR signal change may come from GBCA distribution in both blood and CSF, which is particularly relevant for intravenous GBCA injection, resulting in significant partial volume effects especially when GBCA distribution in the CSF is expected to be limited in healthy human subjects. Besides, most existing human MRI studies on the topic were performed with a temporal resolution of several minutes to a few hours [7, 10, 41], and therefore did not study the initial phase immediately following intravenous GBCA administration.

In this study, we applied the recently developed dynamic susceptibility contrast in the CSF (cDSC) MRI approach [43] to measure GBCA-induced MR signal changes in the CSF around CP and in the lateral ventricle (LV) immediately and 4 h after intravenous administration of a macrocyclic GBCA in healthy subjects. We focused particularly the acute dynamics of GBCA across the BCSFB almost immediately after intravenous administration. This was made possible by a much improved temporal resolution (< 10 s) of the cDSC MRI method compared to typical MRI methods used in similar studies (> 5 min) [44]. Different from the conventional dynamic susceptibility contrast (DSC) MRI that is commonly used to measure GBCA-induced MR signal changes in the blood and parenchyma, cDSC MRI is optimized to measure GBCA-induced MR signal changes *only* from the CSF. The key is to employ a long echo time (TE = 1347 ms) to suppress brain parenchyma and blood signals so that only CSF signal is detected with minimal partial volume effects, as shown in our previous work [43]. GBCA concentration in the CSF can also be estimated from cDSC MRI [43]. Using cDSC MRI, we seek to clarify whether GBCAs can cross the BCSFB in the CP and enter ventricular CSF immediately after intravenous administration in healthy human subjects. The FLAIR MRI sequence is one of the most widely adopted approach in existing studies on GBCA distribution in the human brain [44, 45]. The cDSC MRI results were compared with FLAIR MRI results in the same subjects. The purpose for including the FLAIR sequence is two-fold. First, it provides a link for us to compare the results from the current study with previous works. Second, the partial volume effects from blood when using intravenous GBCAs were compared in FLAIR and cDSC MRI. Finally, the relationship between age and GBCA concentration in the CSF estimated from cDSC MRI was investigated in a cohort of 20–85 years old healthy individuals.

## Methods

### Study participants

In this prospective study conducted from March 2021 to September 2022, a total of 25 healthy volunteers (age 48.9 ± 19.5 years, range 20–85 years; 14 females and 11 males) were recruited for this study. All participants were screened with an estimated glomerular filtration rate (eGFR) > 30 mL/min/1.73m^2^ to be eligible for an MRI scan with GBCA. None of the participants had a history of neurologic or psychiatric disorders. This study was approved by the Johns Hopkins Institutional Review Board, and written informed consent was obtained from each participant.

Among all 25 participants, 10 subjects (age 41.5 ± 17.1 years, range 20–85 years; 6 females and 4 males) completed all MRI scans including the 4-h post-GBCA cDSC and FLAIR MRI scans, and 19 subjects (age 42.5 ± 16.7 years, range 20–85 years; 9 females and 10 males) completed all MRI scans except for the 4-h post-GBCA cDSC MRI scan. Thus, the group sizes for all immediate post-GBCA MRI scans, 4-h post-GBCA FLAIR and cDSC MRI scans were 25, 19, and 10, respectively. This was mainly due to logistic challenges for GBCA studies in healthy human subjects. We note that similar cohort sizes were reported in existing GBCA studies in *healthy* human subjects, for instance, 5 subjects in Kao et al. [35], 9 subjects in Wahlin et al. [41], and 8 subjects in Richmond et al. [10].

### MRI and GBCA contrast

MRI scans were performed on a 3.0 Tesla (3T) Philips human MRI scanner (Philips Healthcare, Best, The Netherlands). A 32-channel phased-array head coil was used for signal reception and a dual-channel body coil for transmit. The GBCA (Gadoteridol (Prohance), 279.3 mg/mL (0.5 mmol/mL) supplied in a single dose; Bracco S.p.A., Milan, Italy) was administered intravenously (i.v.) using a standard procedure (dosage = 0.1 mmol/kg, injection rate = 5 mL/s). The actual GBCA dosage for each subject was calculated based on their body weight.

The following MRI scans were performed for each subject:

1. pre-GBCA FLAIR MRI: repetition time (TR)/inversion time (TI)/echo time (TE) = 6000/2000/180 ms, three dimensional (3D) turbo spin echo (TSE, also known as fast spin echo or FSE) readout, TSE factor = 70, Compressed-Sensing SENSE (CS-SENSE) = 6, voxel = 0.8 × 0.8 × 0.8 mm^3^, 188 slices, scan duration = 8 min and 36 s;
2. cDSC MRI: TR/TE/echo spacing (ES) = 10000/1347/3.2 ms, 3D TSE readout, TSE factor = 1024, single shot, partial Fourier = 0.75(AP) × 0.75(RL), CS-SENSE = 12 (we showed previously that spatial blurring was negligible for CS-SENSE < 16 in the human brain [46]), voxel = 0.8 × 0.8 × 0.8 mm^3^, and 188 slices (same coverage as FLAIR MRI). The sequence was performed continuously with 28 and 32 volumes acquired before and immediately after GBCA injection, respectively. The total scan duration was 10 min and 20 s with two dummy scans in the beginning.
3. post-GBCA FLAIR MRI: identical parameters as pre-GBCA FLAIR MRI were used. This scan was performed immediately after the cDSC MRI scan, which was approximately 6 min after GBCA injection.

In a subset of participants (see Study participants), the following MRI scans were performed at a second visit approximately 4 h after the first MRI session:

4) 4-h post-GBCA FLAIR MRI: identical parameters as pre-GBCA FLAIR MRI were used.
5) 4-h post-GBCA cDSC MRI: voxel = 0.8 × 0.8 × 0.8 mm^3^, 188 slices, scan duration = 3 min and 50 s.

### Simulations

Numerical simulations were performed using the Bloch equations to better interpret the cDSC and FLAIR MRI results. The simulations were coded in Matlab (MathWorks, Natick, MA, USA). The same imaging parameters from the MRI protocol described above were used. Other physical and physiological parameters used in the simulations are summarized in Table 1.

**Table 1:** Literature values for key parameters used in the simulations

	CSF	CSF with GBCA	Blood	Blood with GBCA	GM	WM
*3 T*						
T1 (ms)	4310 ^[54]^	1262^c^	1878^[55]^	199^c^	1607^[56]^	838^[56]^
T2 (ms)	1400^[54]^	717^c^	157^[57], d^	72^c^	63^[58]^	77^[58]^
r1 (L/mmol/s)^a^		2.8^[59]^		4.4^[59]^		
r2 (L/mmol/s)^b^		3.4^[59]^		5.5^[59]^		

^a^  r1 = T1 relaxivity of gadolinium contrast in a certain medium (CSF or blood). It is not field dependent, but changes with the medium

^b^ r2 = T2 relaxivity of gadolinium contrast in a certain medium (CSF or blood). It is not field dependent, but changes with the medium

^c^ The GBCA-induced changes in T1 and T2 relaxation times can be written as:

$$\frac{1}{{T1}_{Gd}}= \frac{1}{{T1}_{0}}+{r}_{1}\times [Gd]$$

$$\frac{1}{{T2}_{Gd}}= \frac{1}{{T2}_{0}}+{r}_{2}\times [Gd]$$

where variables with the subscription “0” (T1_0_ and T2_0_) represent the values before GBCA injection, variables with the subscription “Gd” (T1_Gd_ and T2_Gd_) represent the values after GBCA injection, r1 and r2 are the relaxivity of GBCA in a certain medium (CSF or blood), and [Gd] is the concentration of GBCA in the medium

^d^ In cDSC MRI, we aim to suppress the blood signal using long echo time (TE). Therefore the longest blood T2 values (fully oxygenated blood) were used in the simulations. A typical hematocrit (Hct) level of 0.43 was assumed

### Data analysis

The statistical parametric mapping (SPM) software package (Version 12, Wellcome Trust Centre for Neuroimaging, London, United Kingdom; http://www.fil.ion.ucl.ac.uk/spm/) and other in-house code programmed in Matlab (MathWorks, Natick, MA, USA) were used for image analysis. The cDSC images were motion corrected using the realignment routine in SPM. FLAIR images were co-registered to the mean cDSC images. Masks of cortical gray matter (GM) and white matter (WM) were obtained using the segmentation routine in SPM. Five regions of interest (ROI) were manually delineated and the segmentation was adjusted when needed on the co-registered post-GBCA FLAIR images using ITK-SNAP (Version 3.8; http://www.itksnap.org), which were then overlaid onto all the other MRI scans at all time points from the same subject for further analysis. The five ROIs are: choroid plexus (CP), the transition area between CP and CSF (two layers of voxels around the CP mask within the boundary of ventricle), the rest of lateral ventricle (LV) excluding CP and transition area, cortical GM (cGM) in the occipital lobe, and corpus callosum (CC). All ROI selection was manually performed on all slices by three experienced investigators (Y.S., D.C., and J.J.P.) independently, after which discrepancies among the three investigators were assessed and final selection agreed upon. The volume of LV and intracranial volume (ICV) were calculated for each subject after the segmentation. The average MR signals from the CC was used to normalize MR signals measured from different sessions (pre-GBCA, post-GBCA, and 4 h post-GBCA), as little GBCA induced MR signal change is expected in the CC in healthy subjects during the time period of planned experiments in the current study[45, 47]. The relative signal change (∆S/S) was calculated as the difference between pre- and post-GBCA signals divided by the average pre-GBCA signal in respective MRI scans (cDSC and FLAIR). Spatially, the pre- and post-GBCA signals were average over entire ROIs without voxel selection in respective MRI scans. Temporally, the post-GBCA cDSC signals were averaged over 50–250 s after GBCA injection. The GBCA concentration in the CSF ([Gd]), time to onset (T_onset_), and time to plateau (TTP) were calculated from cDSC MRI using the same methods developed in previous work [43]. Briefly, the change between pre- and post-GBCA CSF signals in cDSC MRI has a monotonic relationship with GBCA concentration, which was exploited to determine [Gd] from ∆S/S [43]. This approach for estimating [Gd] was validated in previous work using GBCA solution phantoms [43], and its potential caveats are described in the Discussion section. These parameters ([Gd], T_onset_, and TTP) cannot be estimated from FLAIR MRI. Note that cDSC MRI is different from conventional DSC MRI, and therefore no DSC software was used in the analysis.

### Statistics

Paired t-tests were used to compare pre-GBCA and post-GBCA measures in each group. The correlations with age from GBCA concentration were evaluated using the Pearson correlation coefficient (r) with the actual GBCA dosage in each subject included as a covariate. Only GBCA concentration estimated from cDSC MRI results were used in the correlation analysis as GBCA concentration cannot be estimated directly from the FLAIR MRI results.

## Results

### Theoretical simulations of MRI signals

**Fig. 1:**
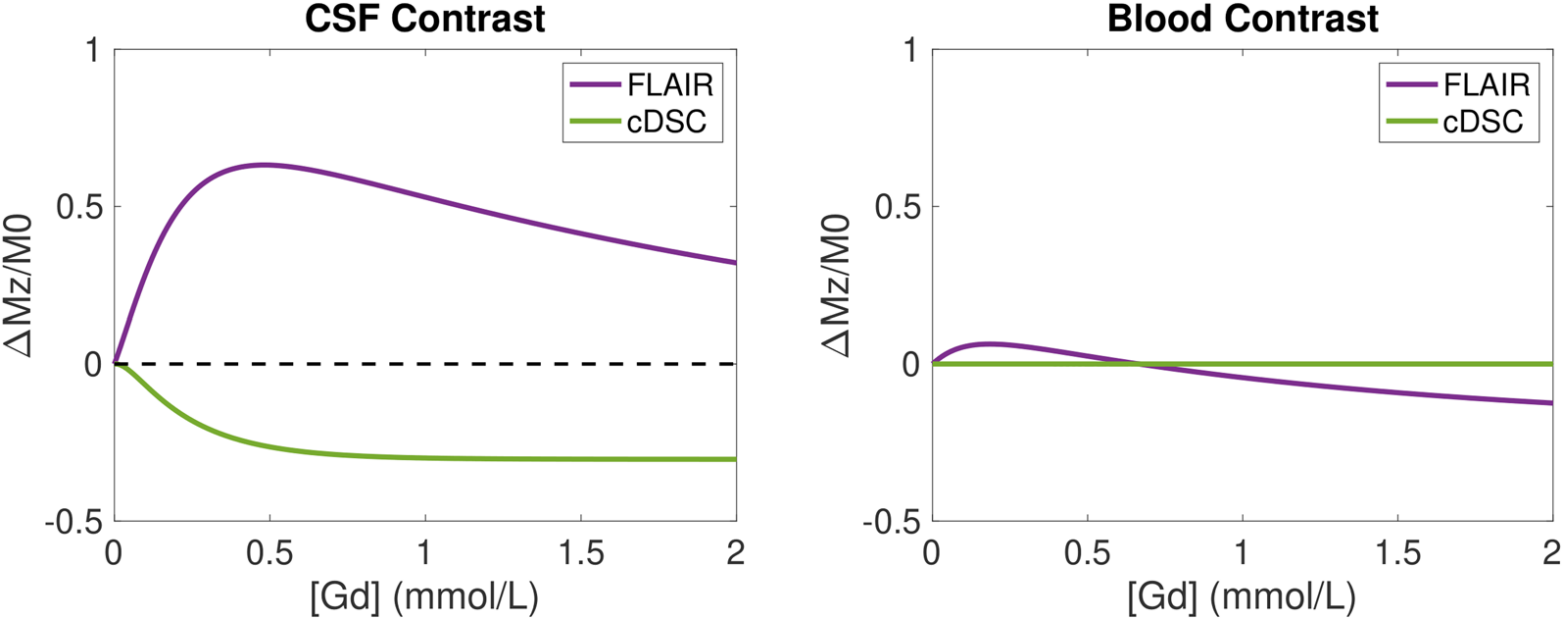
Theoretical simulations of the trend of MRI signals in FLAIR and cDSC MRI. Fractional difference of post versus pre-GBCA magnetization normalized by the equilibrium magnetization (ΔMz/M0) was plotted as a function of GBCA concentration ([Gd], mmol/L) for the FLAIR and cDSC MRI pulse sequences. **a** MRI signal difference in the CSF (CSF Contrast). **b** MRI signal difference in the blood (Blood Contrast). Note that in cDSC MRI, a very long echo time (TE = 1347 ms) was used to suppress blood signal, and therefore the blood contrast in cDSC in the simulations was minimal. FLAIR: fluid-attenuated inversion recovery; cDSC: dynamic-susceptibility-contrast-in-the-CSF; GBCA: gadolinium-based-contrast-agent; CSF: cerebrospinal fluid

The GBCA induced signal changes in the CSF are fundamentally different in the recently developed cDSC MRI and the commonly used FLAIR MRI sequences. First, the FLAIR signals have a strong T1-weighting, which leads to a positive GBCA induced signal change in the CSF (Fig. 1a). The cDSC signals are predominantly T2-weighted, which results in a negative GBCA induced signal change in the CSF [43]. The magnitude of post- versus pre-GBCA signal changes is greater in FLAIR than in cDSC. Second, the cDSC MRI method uses a very long echo time (TE = 1347 ms) so that only CSF signals remain in the images while signals from blood and brain parenchyma are negligible [43]. Thus, the partial volume effect from blood is minimal in cDSC signals. On the other hand, the FLAIR signals include significant contributions from both CSF and blood (Fig. 1b) as well as brain parenchyma in the voxel. Therefore, in the LV where only CSF signals are measured, the magnitude of GBCA induced FLAIR signal changes should show a similar trend as the magnitude of cDSC signal changes (but with opposite signs). Nevertheless, in the CP, transition area, and cGM where partial volume effects from both CSF and blood are substantial, the magnitudes of FLAIR and cDSC signal changes may be opposite. For instance, for [Gd] < 0.5 mmol/L (which is often the case at 4 h after intravenous GBCA administration), an increased GBCA concentration in the CSF but a decreased GBCA concentration in the blood may lead to a greater (more negative) cDSC signal change but a smaller (less positive) FLAIR signal change in the CP, transition area, and cGM, due to substantial partial volume averaging from both blood and CSF signals within the voxel in FLAIR. These simulation results serve as the basis when interpreting subsequent MRI results. Note that a higher GBCA concentration does not always correspond to a greater magnitude of MR signal change, especially in FLAIR MRI.

### Healthy human subject results

**Fig. 2:**
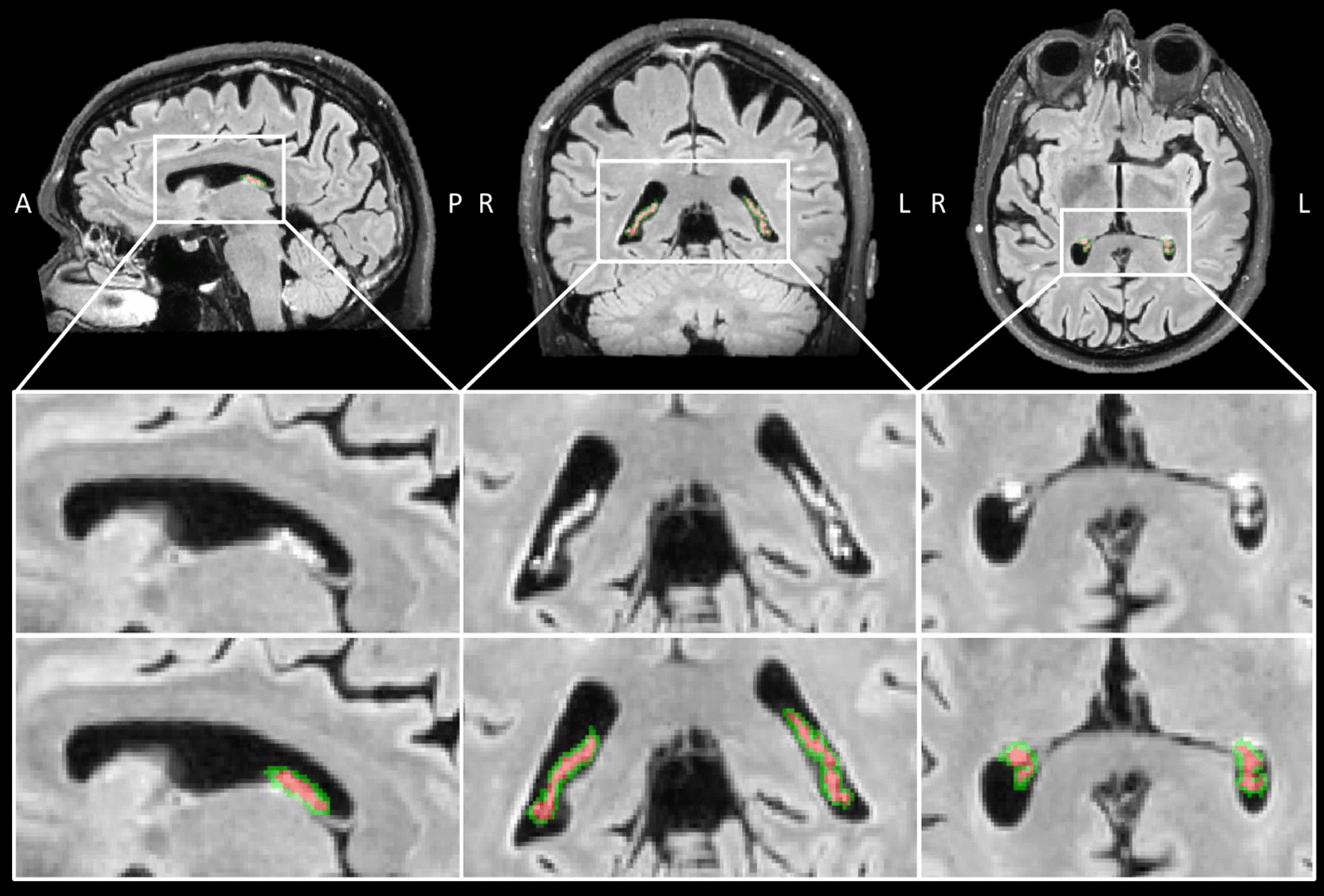
Representative manual segmentation of the choroid plexus (CP, red), and the transition area between the choroid plexus and CSF (green) identified on the post-GBCA FLAIR MRI images (69 years old, male). The regions in the white boxes on the first row are magnified on the second and third rows. The images on the second and third rows are identical with the CP and transition area outlined with red and green, respectively on the third-row images. A: anterior; P: posterior; R: right; L: left. FLAIR: fluid-attenuated inversion recovery; GBCA: gadolinium-based-contrast-agent; CSF: cerebrospinal fluid

Figure 2 demonstrates a representative manual segmentation of the choroid plexus (CP) on the post-GBCA FLAIR images.

**Fig. 3:**
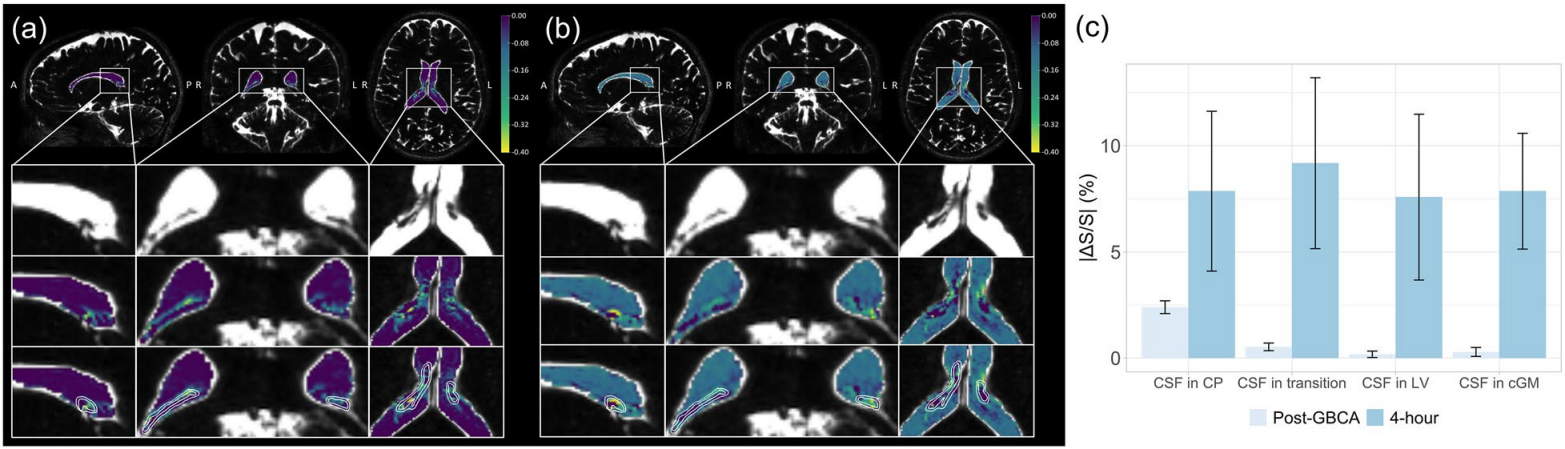
Dynamic signal changes in the CSF around the choroid plexus (CP) measured by cDSC MRI. **a** Representative maps of relative signal changes (ΔS/S) averaged over 50–250 s after intravenous GBCA administration in the lateral ventricle (including the CP) from a healthy human subject (33 years old, female) are shown. Results were overlaid on the pre-GBCA cDSC MRI images. The regions in the white boxes on the first row are magnified on the second to fourth rows. The second row shows the original pre-GBCA cDSC MRI images. The third row displays cDSC MRI images overlaid with ΔS/S in the lateral ventricle. On the fourth row images, the CP and transition area between CP and CSF were outlined with white contours. A: anterior; P: posterior; R: right; L: left. The scale bar indicates the range of ΔS/S. Note that the cDSC MRI method has a negative contrast for GBCA-induced signal changes in the CSF. **b** Representative maps of ΔS/S approximately 4 h after intravenous GBCA administration in the lateral ventricle (including the CP) from the same healthy human subject are shown. Results were overlaid on the pre-GBCA cDSC MRI images. **c** Average absolute ΔS/S in the CSF immediately and 4 h after intravenous GBCA administration measured by cDSC MRI from all healthy human subjects (n = 25). The CSF signal changes in four ROIs were calculated: the choroid plexus (CP), transition area between CP and CSF (transition), the rest of lateral ventricle (LV), and the perivascular space of occipital cortical grey matter (cGM). The error bars represent inter-subject standard errors. Quantitative results are reported in Tables 2 and 3. Note that absolute ΔS/S is displayed here for easier comparison with subsequent results. cDSC: dynamic-susceptibility-contrast-in-the-CSF; GBCA: gadolinium-based-contrast-agent; CSF: cerebrospinal fluid

**Fig. 4:**
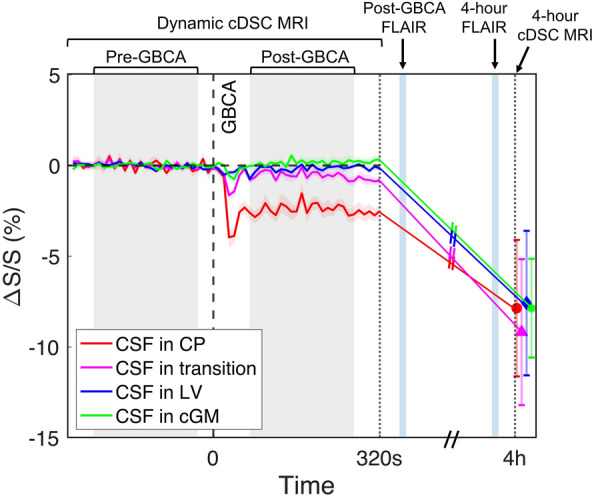
Average time courses of the dynamic signal changes in the CSF (ΔS/S) before and after intravenous GBCA administration measured by the cDSC MRI approach from all healthy human subjects (n = 25) are displayed. The CSF signal changes in four ROIs were calculated: the choroid plexus (CP), transition area between CP and CSF (transition), the rest of lateral ventricle (LV), and the perivascular space of occipital cortical grey matter (cGM). The shaded areas around the time courses represent inter-subject standard errors. The vertical dashed lines indicate the time when GBCA was injected. The shaded areas indicate the pre- and post-GBCA periods over which ΔS/S was averaged and reported in Table 2. The first vertical dotted line indicates the end of the dynamic cDSC MRI scans. The second vertical dotted line indicates the time when the follow-up dynamic cDSC scan (4 h after GBCA injection) was performed. The light blue vertical lines indicate when the FLAIR MRI scans were performed. FLAIR: fluid-attenuated inversion recovery; cDSC: dynamic-susceptibility-contrast-in-the-CSF; GBCA: gadolinium-based-contrast-agent; CSF: cerebrospinal fluid

Significant signal changes were detected from the CSF around the CP in cDSC MRI images in all healthy subjects after intravenous GBCA administration. Figure 3a and b show a representative map of relative signal changes (ΔS/S) measured using cDSC MRI in the lateral ventricle (including CP) a few minutes (averaged over 50–250 s) and 4 h post-GBCA, respectively. Note that the cDSC MRI approach has a negative contrast for GBCA-induced signal changes in the CSF. The time courses from each ROI averaged over all subjects are shown in Fig. 4 and the quantitative results are summarized in Fig. 3c, and Tables 2 and 3. In the CSF around the CP and in the transition area, significant signal changes were detected using cDSC MRI approximately 20 s (onset time, or T_onset_) after intravenous GBCA injection, which reached a plateau at approximately 30 s (time to plateau, or TTP) post-GBCA, and persisted for the rest of the cDSC MRI scan (5 min). During the period of 50–250 s post-GBCA, significant cDSC signal changes were observed only in the CSF around the CP and in the transition area, but not in the rest of lateral ventricle (LV) and in the perivascular space of cortical grey matter (cGM). The GBCA concentration in the CSF around the CP and in the transition area during this period estimated from cDSC images was in the range of 0.01–0.03 mmol/L. At 4 h after intravenous GBCA administration, cDSC images showed significant signal changes compared to pre-GBCA signals in all four ROIs, including the CSF around the CP, in the transition area, in the rest of the LV, and in the perivascular space of cGM. The magnitudes of relative signal changes at 4 h in all ROIs were greater compared to those during the 50–250 s post-GBCA period, respectively. The GBCA concentration in the CSF around the CP was significantly (P < 0.05) greater compared to that in the rest of the LV during the 50–250 s post-GBCA period, but became comparable (P > 0.1) at 4 h.

**Table 2:** Quantitative results measured by dynamic cDSC MRI immediately after intravenous GBCA administration (Post-GBCA period)^a^ from all healthy human subjects (n = 25)

	ΔS/S (%) ^b^	P (vs. pre-GBCA)	[Gd] (mmol/L) ^c^	T_onset_ (s)	TTP (s)
CSF around CP	− 2.40 ± 0.30	< 0.001*	0.0266 ± 0.0019	20 ± 6	32 ± 19
CSF in Transition	− 0.53 ± 0.18	0.01*	0.0125 ± 0.0019	20 ± 6	30 ± 19
Rest of LV	− 0.18 ± 0.15	0.24	0.0079 ± 0.0017	N/A ^d^	N/A ^d^
CSF around cGM	0.29 ± 0.21	0.17	N/A	N/A ^d^	N/A ^d^

CP: choroid plexus; Transition: the transition area between CP and CSF; LV: lateral ventricle; cGM: cortical grey matter in the occipital lobe; T_onset_: onset time; TTP: time to plateau; all values: mean ± standard error (inter-subject)

^a^ The post-GBCA period is 50–250 s for cDSC MRI

^b^ Relative change ΔS/S was defined as 100 * (mean post-GBCA—mean pre-GBCA)/(mean pre-GBCA) %

^c^ [Gd] = concentration of GBCA

^d^ T_onset_ and TTP were not estimated when ΔS/S was not significant

**Table 3:** Quantitative results measured by cDSC MRI at 4 h after intravenous GBCA administration (4 h post-GBCA) from 10 healthy human subjects (n = 10)

	Post-GBCA period (n = 10) ^a^					4 h post-GBCA (n = 10)			
	ΔS/S (%) ^b^	P (vs. pre- GBCA)	[Gd] ^c^ (mmol/L)	T_onset_ (s)	TTP (s)	ΔS/S (%) ^b^	P (vs. pre- GBCA)	[Gd] ^c^ (mmol/L)	P (vs. post- GBCA)
CSF around CP	− 1.54 ± 0.31	0.001*	0.0212 ± 0.0029	24 ± 10	34 ± 24	− 7.86 ± 3.76	0.03*	0.0542 ± 0.0112	0.06
CSF in Transition	0.01 ± 0.22	0.98	0.0075 ± 0.0026	24 ± 11	36 ± 28	− 9.18 ± 4.02	0.02*	0.0578 ± 0.0126	0.02*
Rest of LV	− 0.09 ± 0.14	0.51	0.0079 ± 0.0023	N/A ^d^	N/A ^d^	− 7.58 ± 3.9	0.04*	0.0501 ± 0.0130	0.04*
CSF around cGM	0.47 ± 0.32	0.17	N/A	N/A ^d^	N/A ^d^	− 7.86 ± 2.72	0.02*	0.0485 ± 0.0105	0.003*

CP: choroid plexus; Transition: the transition area between CP and CSF; LV: lateral ventricle; cGM: cortical grey matter in the occipital lobe; T_onset_: onset time; TTP: time to plateau; all values: mean ± standard error (inter-subject)

^a^ The post-GBCA period is 50–250 s for cDSC MRI. Among all 25 participants, 10 subjects completed the 4-h post-GBCA cDSC MRI scan

^b^ Relative change ΔS/S was defined as 100 * (mean post-GBCA – mean pre-GBCA) / (mean pre-GBCA) %

^c^ [Gd] = concentration of GBCA

^d^ T_onset_ and TTP were not estimated when ΔS/S was not significant

**Fig. 5:**
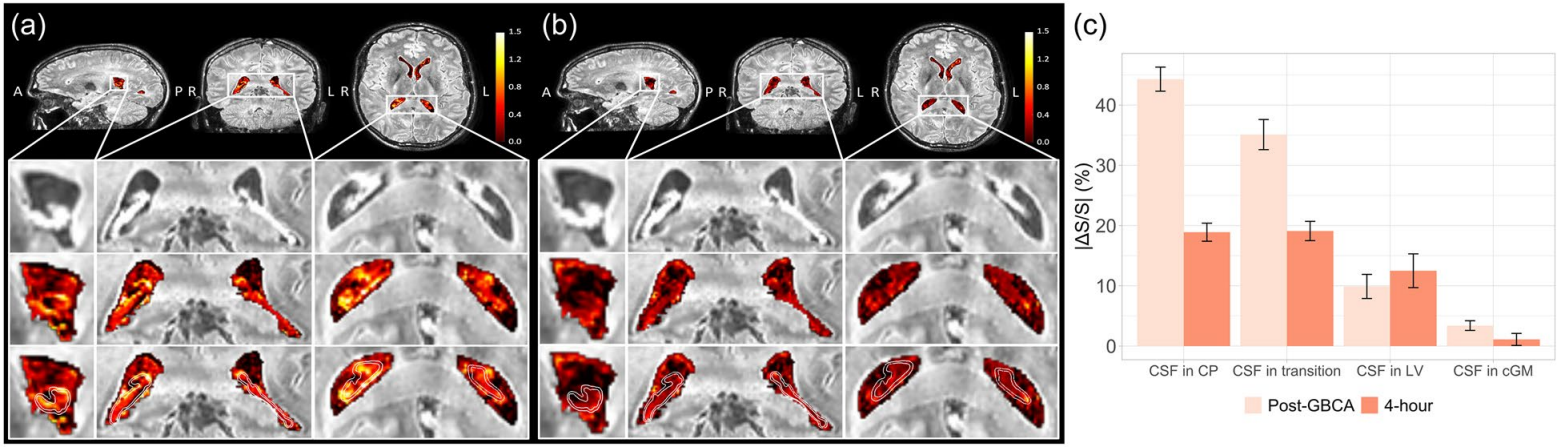
GBCA induced signal changes in the choroid plexus (CP) measured by FLAIR MRI. **a** Representative maps of relative signal changes (ΔS/S) in the lateral ventricle (including the CP) approximately 6 min after intravenous GBCA administration from a healthy human subject (31 years old, female) are shown. The signal changes were overlaid on the post-GBCA FLAIR MRI images. The scale bar indicates the range of ΔS/S. Note that the FLAIR MRI method has a positive contrast for GBCA-induced signal changes in the CSF. The regions in the white boxes on the first row are magnified on the second to fourth rows. The images on the second to fourth rows are identical anatomically with the CP and transition area between CP and CSF outlined with white contours on the fourth row images. A: anterior; P: posterior; R: right; L: left. **b** Representative maps of relative signal changes (ΔS/S) in the lateral ventricle (including the CP) approximately 4 h after intravenous GBCA administration from the same healthy human subject are shown. The signal changes were overlaid on the post-GBCA FLAIR MRI images. **c** Average absolute ΔS/S immediately and 4 h after intravenous GBCA administration measured by FLAIR MRI from all healthy human subjects (n = 25). The signal changes in four ROIs were calculated: the choroid plexus (CP), transition area between CP and CSF (transition), the rest of lateral ventricle (LV), and the perivascular space of occipital cortical grey matter (cGM). The error bars represent inter-subject standard errors. Quantitative results are reported in Table 4. FLAIR: fluid-attenuated inversion recovery; GBCA: gadolinium-based-contrast-agent; CSF: cerebrospinal fluid

Significant signal changes were also detected from the CP in FLAIR MRI images in all healthy subjects after intravenous GBCA administration. Figure 5a and 5b show a representative map of relative signal changes (ΔS/S) measured using FLAIR MRI in the lateral ventricle (including CP) approximately 6 min and 4 h post-GBCA, respectively. Note that the FLAIR MRI method has a positive contrast for GBCA-induced signal changes in the CSF. The quantitative results are summarized in Fig. 5c and Table 4. At 6 min post-GBCA, significant FLAIR signal changes were observed in the CP and transition area, but not in the rest of lateral ventricle (LV). At 4 h post-GBCA, FLAIR images showed significant signal changes compared to pre-GBCA signals in the CP, transition area, and LV, but not in the cGM. The relatively signal changes at 4 h in the CP and transition area were both smaller compared to those at 6 min post-GBCA, whereas the relative signal changes in the LV showed an increasing trend compared to those at 6 min post-GBCA.

**Table 4:** Quantitative results measured by FLAIR MRI from all healthy human subjects

	Post-GBCA period ^a^		4 h post-GBCA		
	ΔS/S (%) ^b^	P (vs. pre-GBCA)	ΔS/S (%) ^b^	P (vs. pre-GBCA)	P (vs. post-GBCA ΔS/S)
	(n = 25) ^c^		(n = 19) ^c^		
CSF around CP	44.3 ± 2.0	< 0.001*	18.9 ± 1.5	< 0.001*	< 0.001*
CSF in Transition	35.1 ± 2.5	< 0.001*	19.1 ± 1.6	< 0.001*	< 0.001*
Rest of LV	9.9 ± 2.0	0.15	12.5 ± 2.8	0.01*	0.14
CSF around cGM	3.4 ± 0.8	0.001*	1.1 ± 1.0	0.67	0.11

CP: choroid plexus; Transition: the transition area between CP and CSF; LV: lateral ventricle; cGM: cortical grey matter in the occipital lobe; all values: mean ± standard error (inter-subject)

^a^ The post-GBCA period is 5–10 min for FLAIR MRI

^b^ Relative change ΔS/S was defined as 100 * (mean post-GBCA – mean pre-GBCA) / (mean pre-GBCA) %

^c^ Among all 25 participants, 19 subjects completed the 4-h post-GBCA FLAIR MRI scan

Note that GBCA concentration ([Gd]), T_onset_ and TTP cannot be estimated from FLAIR MRI

### Correlation with age from GBCA concentration

**Fig. 6:**
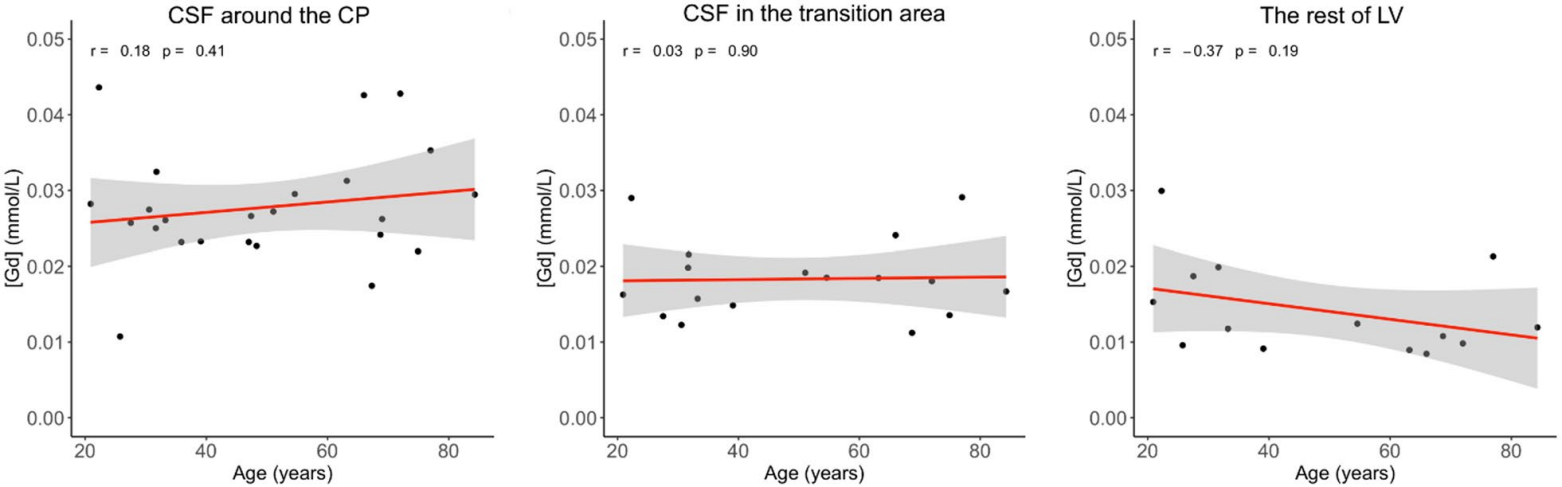
Correlation with age from GBCA concentration in the CSF around the choroid plexus (CP), transition area and the rest of lateral ventricle (LV) during the 50–250 s post-GBCA period in all subjects (n = 25) while controlling for GBCA dosage in each subject (based on body weight). r: Pearson correlation coefficient. GBCA: gadolinium-based-contrast-agent; CSF: cerebrospinal fluid

**Fig. 7:**
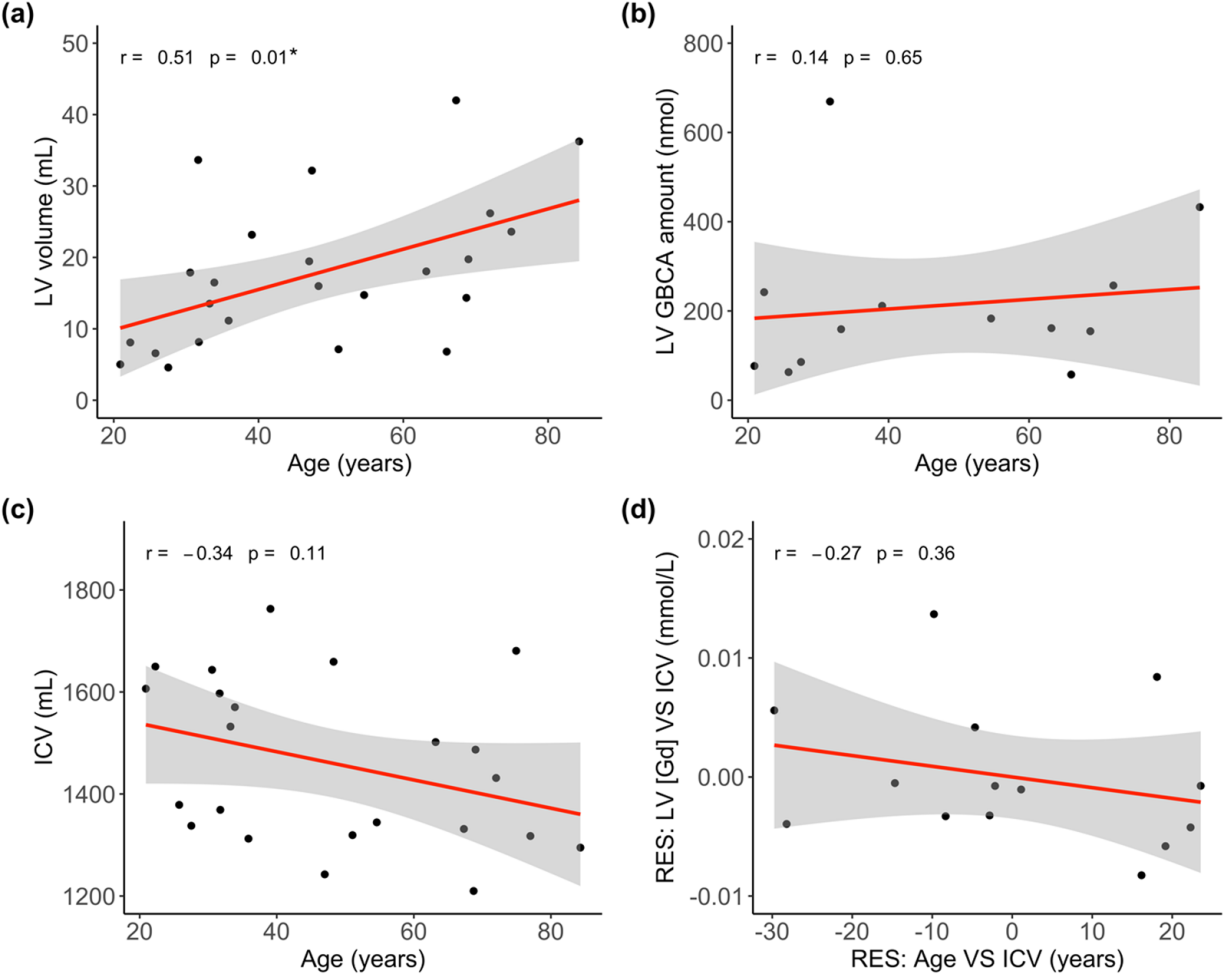
Correlation analysis with the volume of lateral ventricle (LV) and intracranial volume (ICV) in all subjects (n = 25). **a** Correlation between age and the volume of LV. **b** Correlation between age and GBCA amount in the LV (= GBCA concentration × volume of LV). GBCA concentration in the LV during the 50–250 s post-GBCA period was used. **c** Correlation between age and ICV. **d** Partial correlation between age and GBCA concentration ([Gd]) in the LV with ICV as a covariate. The x-axis and y-axis are residuals from the correlation between age and ICV, and from the correlation between LV [Gd] and ICV, respectively. GBCA concentration in the LV during the 50–250 s post-GBCA period was used. r: Pearson correlation coefficient. GBCA: gadolinium-based-contrast-agent; CSF: cerebrospinal fluid

As shown in Fig. 6, the GBCA concentration ([Gd]) in the CSF around the CP, the transition area and the rest of LV estimated from cDSC MRI images during the 50–250 s post-GBCA period did not show significant correlation with age when controlling for GBCA dosage in each subject (proportional to body weight). Figure 7 shows the correlation results when effects from the volume of LV and ICV are accounted for. A significant correlation between age and the volume of LV was observed (p = 0.01). The amount of GBCA in the LV, which was calculated as the produce of GBCA concentration in the LV and volume of LV, did not show significant correlation with age. The ICV did not correlate with age in our cohort. When the ICV was accounted for as a covariate, GBCA concentration in the LV did not show significant correlation with age.

## Discussion

Our results provided evidence that a commonly used macrocyclic GBCA can cross the BCSFB in the CP and enter CSF circulation after intravenous injection even in *healthy* human subjects. Two MRI pulse sequences, FLAIR and cDSC MRI were performed to measure GBCA induced signal changes in the CSF around CP and in the LV immediately and 4 h after intravenous GBCA administration. The FLAIR MRI approach has been widely used in previous studies [44, 45]. While the temporal resolution of FLAIR is usually lower than cDSC MRI, the inversion recovery technique used in FLAIR can produce a stronger GBCA-induced CSF signal change than cDSC MRI (see simulations in Fig. 1a). The cDSC MRI approach is a recently developed sequence that can measure GBCA-induced CSF signal changes with a temporal resolution of < 10 s and a sub-millimeter spatial resolution in the entire brain [43]. It employs a long TE (1347 ms) to suppress blood and brain parenchyma signals with typical T2 relaxation times shorter than 150 ms. As demonstrated in the original technical work [43], this approach provides clean CSF signals even when the actual MRI voxel includes blood vessels and parenchyma. Therefore, the major difference between the GBCA-induced signal changes measured by cDSC and FLAIR is that the cDSC signals can be confidently interpreted as pure CSF signals, but the overall signal in a voxel in FLAIR images can have substantial contributions from all compartments (blood, CSF, and parenchyma). Indeed in our human MRI results, in the LV where partial volume effects from blood and parenchyma are minimal, the trends of cDSC and FLAIR signal changes agreed well, with greater magnitudes at 4 h compared to the 50–250 s post-GBCA period from both methods. Nevertheless, in the CP, transition area, and cGM where partial volume effects from blood are substantial, the magnitude of ΔS/S became smaller in FLAIR but greater in cDSC at 4 h compared to the 50–250 s post-GBCA period. According to our simulations, this could be a result of increased GBCA concentration in the CSF but decreased GBCA concentration in the blood at 4 h. Such partial volume effects from blood, CSF, and parenchyma in a voxel is an important caveat that should be cautioned when interpreting intravenous GBCA enhanced MRI results using FLAIR and other pulse sequences that have signal contributions from both blood and CSF.

With a 10 s temporal resolution provided by cDCS MRI, the onset time (T_onset_) of GBCA-induced signal changes in the CSF around CP after intravenous administration was estimated to be about 20 s, which was comparable to the T_onset_ reported for meningeal lymphatic vessels in the parasagittal dura region in healthy human subjects [43]. The time to plateau (TTP) in the CSF around CP (about 30 s) was slightly shorter than the TTP reported for meningeal lymphatic vessels (approximately 50 s) in the parasagittal dura region in healthy human subjects [43]. There is little literature on such temporal characteristics for intravenous GBCAs, as most existing studies were performed with a temporal resolution of a few minutes. In brain tumor patients, similar T_onset_ and TTP were measured in brain tissue using dynamic contrast enhancement (DCE) MRI after intravenous GBCAs leak out from the broken BBB in the tumor region [48, 49]. It is worth noticing that the time courses measured from the CSF around the CP, in the transition area, and surrounding cortical GM in our data all showed a dip around 20–30 s post-GBCA. The timing coincides with the initial bolus phase for GBCAs in the blood after intravenous injection. Therefore, the dip can be caused by the initial rush of high concentration GBCAs from the blood during the bolus phase. After that, the blood GBCA concentration becomes much lower, and the GBCAs that have entered CSF begin to dissipate into the surrounding CSF space. Note that the time course measured from the LV did not show such a dip. However, various other factors can also result in such a dip, such as head motion during this period. In this study, head motion was restricted by careful padding and motion correction was performed for all images. But residual motion especially in small areas such as the CP can still affect the time courses. The origin of this signal dip during the initial phase warrants further investigation. In our analysis, this initial phase was excluded when calculating ΔS/S.

During the 50–250 s post-GBCA period, significant GBCA-induced signal changes were detected in the CSF around CP, but not in the rest of the LV. At 4 h post-GBCA, significant GBCA-induced signal changes remained in the CSF around CP, and were also detected in the rest of the LV. Therefore, our data indicate that the GBCAs entered CSF via the CP in about 20 s after intravenous injection, and then gradually spread across the LV over time. At 4 h, significant GBCA-induced signal changes were also observed in the perivascular space of occipital cGM. The timing of GBCA-induced CSF signal changes in these regions is consistent with previous studies [7, 10, 41]. In the study by Deike-Hofmann et al. [7], significant GBCA-induced signal changes were detected in the CP, LV and perivascular space at 3 h after intravenous injection. In the study by Wahlin et al. [41], significant GBCA-induced signal changes were detected in the LV and subarachnoid space 30 min after intravenous injection, and peaked around 2.5 h post-GBCA. In the study by Richmond et al. [10], significant GBCA-induced signal changes were detected in the CP, LV and cortex from 30 min after intravenous injection, and the magnitude in the CP dropped at 3 h post-GBCA. The eventual distribution of GBCA in brain parenchyma was also reported in other studies in healthy individuals [50].

Complete clearance of GBCA was not observed in the CP, LV and cGM at 4 h in our data. Previous studies performed using inductively coupled plasma mass spectrometry (ICP-MS) in human CSF samples obtained from lumbar puncture reported that GBCAs can still be detectable in the CSF and serum of patients for up to 48 h [12] to 24 days [13] after intravenous injection. Subsequent studies conducted over a longer time period than 4 h post-GBCA are merited to further investigate the clearance of GBCAs from the CSF and brain in healthy subjects.

GBCA concentration in the CSF can be estimated from the measured cDSC signal changes as described in our previous work [43]. According to DSC and DCE MRI literature, the typical average GBCA concentration in blood after the bolus phase following intravenous injection with a standard dose is ~ 1 mmol/L in the human brain [51]. During the 50–250 s post-GBCA period, GBCA concentration was in the range of 0.01–0.03 mmol/L in the CSF around CP (i.e. ~ 1–3% of blood GBCA concentration), and was negligible in the other ROIs. At 4 h post-GBCA, GBCA concentration in all ROIs were in the range of 0.05–0.06 mmol/L (i.e. ~ 5–6% of blood GBCA concentration), including the LV and the perivascular space of occipital cGM. Comparing to blood GBCA concentration, this range of GBCA concentration in the CSF appears to be reasonable. Previous studies using ICP-MS reported a GBCA concentration of 1152 ± 735 ng/mL in healthy human CSF samples obtained from lumbar puncture 8 h after intravenous GBCA administration. In our results, the GBCA concentration in the LV at 4 h post-GBCA was equivalent to 27986 ± 7262 ng/mL (converted based on the concentration of Gadoteridol (Prohance) used in this study: 279.3 mg/mL (0.5 mmol/mL)), which is much higher than the ICP-MS results. A main difference may be that the GBCA concentration in the spine can be much lower than the LV; and at 8 h, more GBCAs may have been cleared from the CSF compared to 4 h (current study). A recent study by Wahlin et al. [41] (in conference abstract form) reported a ~ 0.004 mmol/L GBCA concentration in the LV in 9 older healthy human subjects (age 76 ± 4 years) at 2.5–3 h after intravenous GBCA injection using a T1 mapping MRI approach, which is about an order lower than the values estimated in our study. Several factors might contribute to such difference, including a cohort of different age, different MRI methods, and the type of GBCA used. Nevertheless, the calculation of GBCA concentration from MR measures needs to be carefully validated, which is a complicated topic that has been investigated extensively in DSC and DCE MRI for more than 30 years [51]. The GBCA concentration estimated from cDSC MRI was validated using phantoms made with solutions of water and GBCAs of various concentration levels [43]. However, the microenvironment in vivo can be much more complicated and key assumptions such as a dilute aqueous solution of GBCA may not be proper in some regions. Furthermore, literature values were assumed for several parameters in the equations when calculating GBCA concentration, such as CSF T1 and T2, and r1 and r2 of the GBCA in CSF (Table 1) [43]. Table S1 in the Supplemental Material demonstrates how variations in these parameters can affect GBCA concentration calculation in cDSC MRI. For instance, when using a 20% shorter T1, a 20% lower r1, and a 20% higher r2 than the currently assumed values, GBCA concentration in the LV measured at 4 h post-GBCA would become ~ 0.02 mmol/L. Finally, although the partial volume effects from GBCA induced blood signal changes are minimal in cDSC MRI, intravoxel averaging can still introduce errors for estimating GBCA concentration in small regions such as the CP, which can only be improved by imaging at a higher spatial resolution. Subsequent studies are warranted to further validate the accuracy of the GBCA concentration results. However, the trend of GBCA concentration change at 4 h compared to 50–250 s post-GBCA was clearly demonstrated in the current study.

The BCSFB in the CP may be affected by aging and various diseases. The GBCA concentration in ventricular CSF after intravenous injection may be a useful indicator for the integrity of BCSFB. Interestingly, no significant correlation with age from the GBCA concentration in the CP and LV was found in this healthy cohort, suggesting that the CP’s permeability to this GBCA may be largely preserved in normal aging. Nevertheless, one limitation of the current study is that permeability measures are highly dependent on the tracers used, and caution should be taken when interpreting the results using one particular type of tracer alone. Besides, the correlation analysis was only performed with GBCA concentration measured during the initial blood to CSF phase (50–250 s post-GBCA). Unfortunately, only 10 subjects completed the cDSC MRI scan at 4 h after GBCA administration in this study, which were not sufficiently powered for the correlation analysis. The GBCA concentration measured a few hours after intravenous injection reflects primarily the clearance of GBCA from the brain, which may serve as a useful marker for delayed clearance of abnormal proteins in aging and neurodegenerative diseases [19].

It is worth noting that the human results in this study demonstrated some key differences from results obtained from animal studies [19]. For instance, GBCA-induced signal changes in the entire LV were barely detectable in our human data following intravenous injection. However, even in wild type rodents, significant GBCA-induced signal changes are usually observed in the entire LV instantaneously following intravenous GBCA injection [19], when the same dosage was used (standard human GBCA dose calibrated by animal body weight [52]). Many factors can contribute to such differences, for example, the volume of LV is much smaller and the CSF turnover is much faster in rodents than in humans, and the permeability of BCSFB to GBCAs can be very different between rodents and humans. This stresses the importance of validating findings from animal models in healthy human subjects for GBCA-based studies on brain CSF circulation and clearance.

There are a few limitations in the current study which should be addressed in subsequent studies. First, only the CP in the LV was investigated here. Our results certainly do not exclude other potential GBCA entry points into the CSF. While the CP in the LV is considered a main site for CSF production in humans, the CP in the other ventricles, cisterns [9, 53], and the circumventricular organs (CVO) are also believed to contribute to the GBCA permeability between blood vessels and CSF circulation in the human brain [14]. These structures are even smaller than the CP in the LV, which requires additional expertise in neuroradiology to accurately identify them on structural MRI images. We are currently working with a group of experienced neuroradiologists to study GBCA induced signal changes in these regions. Second, only a sub-group of participants completed the MRI scans at 4 h after GBCA administration, and no MRI scans were performed beyond 4 h. The main reason is that this is logistically difficult to arrange especially when GBCA administration is involved in healthy human subjects. Fortunately, GBCA-induced MRI signal changes in the target regions were much greater at 4 h post-GBCA compared to immediate post-GBCA, allowing the detection of significant group effects with a cohort of 10 subjects. Although this group size (10 subjects) was still comparable to existing GBCA studies in *healthy* human subjects [10, 35, 41], in subsequent studies, we will attempt to replicate our findings in a larger cohort and to track the GBCA-induced signal changes for an extended period after GBCA administration to further investigate its distribution in the brain and clearance from the brain.

## Conclusions

In summary, using the cDSC MRI approach developed to measure GBCA-induced MR signal changes *only* from the CSF, our results showed direct imaging evidence that a commonly used macrocyclic GBCA can pass the BCSFB in the CP and enter the CSF circulation via the ventricle immediately after intravenous administration in healthy human brains. The methodology and results obtained in this healthy human study established a useful basis for subsequent studies on BCSFB in the CP and CSF circulation and clearance in aging and various brain diseases. Although many human MRI studies on the topic used intrathecally administrated GBCAs, intravenous administration remains the most widely adopted and practical procedure for GBCA administration in human subjects in the clinics. Therefore, a better understanding of the pathways taken by intravenous GBCAs has high clinical significance.

## Supplementary Information

Additional file 1

## Data Availability

The data that support the findings of this study are available on request from the corresponding author, J.H. The data are not publicly available due to privacy restrictions of research participant**. **No datasets were generated or analysed during the current study.

## Abbreviations

GBCA: Gadolinium-based contrast agent
CSF: Cerebrospinal fluid
BCSFB: Blood-CSF-barrier
CP: Choroid plexus
BBB: Blood–brain barrier
LV: Lateral ventricle
ICP-MS: Inductively coupled plasma mass spectrometry
FLAIR: Fluid-attenuated inversion recovery
cDSC: Dynamic susceptibility contrast in the CSF
DSC: Dynamic susceptibility contrast
TSE: Turbo spin echo
CS-SENSE: Compressed-Sensing SENSE
ROI: Regions of interest
cGM: Cortical gray matter
CC: Corpus callosum
∆S/S: Relative signal change
[Gd]: GBCA concentration in the CSF
T_onset_: Time to onset
TTP: Time to plateau
DCE: Dynamic contrast enhancement
