# Supplementary material for: Rapid imaging of intravenous gadolinium-based contrast agent (GBCA) entering ventricular cerebrospinal fluid (CSF) through the choroid plexus in healthy human subjects
Source: Fluids Barriers CNS. 2024 Sep 16;21:72. doi: 10.1186/s12987-024-00571-3 (PMC11403782; doi:10.1186/s12987-024-00571-3)
Supplement: Supplementary file 1 — Additional file 1 [file 12987_2024_571_MOESM1_ESM.docx]

**Supplementary Table 1S:** Simulation results to demonstrate how variations in CSF T1 and T2 values and GBCA r1 and r2 values can affect GBCA concentration calculation in cDSC MRI. The current values were adopted from the literature (**Table 1**). Results in this table show how variations in these parameters can lead to variations in the estimated GBCA concentration from the same relative signal changes measured in cDSC MRI (assuming ΔS/S = -3% or -7% as examples).

|  | **CSF T1** | | | | |
| --- | --- | --- | --- | --- | --- |
| T1 values (ms) | 2155 | 3448 | 4310 | 5172 | 6465 |
| Relative change from the current value | -50% | -20% | 0%  (current value) | +20% | +50% |
| **ΔS/S in cDSC MRI** | **GBCA concentration (mmol/L)**  estimated from ΔS/S using respective T1 values | | | | |
| **-3%** | 0.009 | 0.017 | 0.032 | 0.055 | 0.088 |
| **-7%** | 0.020 | 0.033 | 0.049 | 0.070 | 0.101 |
|  |  |  |  |  |  |
|  | **CSF T2** | | | | |
| T2 values (ms) | 700 | 1120 | 1400 | 1680 | 2100 |
| Relative change from the current value | -50% | -20% | 0%  (current value) | +20% | +50% |
| **ΔS/S in cDSC MRI** | **GBCA concentration (mmol/L)**  estimated from ΔS/S using respective T2 values | | | | |
| **-3%** | 0.032 | 0.032 | 0.032 | 0.032 | 0.032 |
| **-7%** | 0.049 | 0.049 | 0.049 | 0.049 | 0.049 |
|  |  |  |  |  |  |
|  | **GBCA r1 in CSF** | | | | |
| r1 values (L/mmol/s) | 1.4 | 2.24 | 2.8 | 3.36 | 4.2 |
| Relative change from the current value | -50% | -20% | 0%  (current value) | +20% | +50% |
| **ΔS/S in cDSC MRI** | **GBCA concentration (mmol/L)**  estimated from ΔS/S using respective r1 values | | | | |
| **-3%** | 0.015 | 0.024 | 0.032 | 0.039 | 0.046 |
| **-7%** | 0.031 | 0.043 | 0.049 | 0.055 | 0.060 |
|  |  |  |  |  |  |
|  | **GBCA r2 in CSF** | | | | |
| r2 values (L/mmol/s) | 1.7 | 2.72 | 3.4 | 4.08 | 5.1 |
| Relative change from the current value | -50% | -20% | 0%  (current value) | +20% | +50% |
| **ΔS/S in cDSC MRI** | **GBCA concentration (mmol/L)**  estimated from ΔS/S using respective r2 values | | | | |
| **-3%** | 0.105 | 0.050 | 0.032 | 0.022 | 0.013 |
| **-7%** | 0.128 | 0.069 | 0.049 | 0.037 | 0.025 |
